# Controllable synthesis of molybdenum tungsten disulfide alloy for vertically composition-controlled multilayer

**DOI:** 10.1038/ncomms8817

**Published:** 2015-07-23

**Authors:** Jeong-Gyu Song, Gyeong Hee Ryu, Su Jeong Lee, Sangwan Sim, Chang Wan Lee, Taejin Choi, Hanearl Jung, Youngjun Kim, Zonghoon Lee, Jae-Min Myoung, Christian Dussarrat, Clement Lansalot-Matras, Jusang Park, Hyunyong Choi, Hyungjun Kim

**Affiliations:** 1 School of Electrical and Electronic Engineering, Yonsei University, Seoul 120-749, Korea; 2 School of Materials Science and Engineering, Ulsan National Institute of Science and Technology (UNIST), Ulsan 689-798, Korea; 3 Department of Materials Science and Engineering, Yonsei University, Seoul 120-749, Korea; 4 Air Liquide Laboratories, Wadai 28, Tsukuba, Ibaraki 300-4247, Japan; 5 Air Liquide Laboratories Korea, Yonsei Engineering Research Park, 50 Yonsei-ro, Seodaemun-gu, Seoul 120-749, Korea

## Abstract

The effective synthesis of two-dimensional transition metal dichalcogenides alloy is essential for successful application in electronic and optical devices based on a tunable band gap. Here we show a synthesis process for Mo_1−*x*_W_*x*_S_2_ alloy using sulfurization of super-cycle atomic layer deposition Mo_1−*x*_W_*x*_O_*y*_. Various spectroscopic and microscopic results indicate that the synthesized Mo_1−*x*_W_*x*_S_2_ alloys have complete mixing of Mo and W atoms and tunable band gap by systematically controlled composition and layer number. Based on this, we synthesize a vertically composition-controlled (VCC) Mo_1−*x*_W_*x*_S_2_ multilayer using five continuous super-cycles with different cycle ratios for each super-cycle. Angle-resolved X-ray photoemission spectroscopy, Raman and ultraviolet–visible spectrophotometer results reveal that a VCC Mo_1−*x*_W_*x*_S_2_ multilayer has different vertical composition and broadband light absorption with strong interlayer coupling within a VCC Mo_1−*x*_W_*x*_S_2_ multilayer. Further, we demonstrate that a VCC Mo_1−*x*_W_*x*_S_2_ multilayer photodetector generates three to four times greater photocurrent than MoS_2_- and WS_2_-based devices, owing to the broadband light absorption.

## Abstract (web summary)

The band gap modulation of two-dimensional transition metal dichalcogenide alloy is essential for successful applications. Here, we show a controllable synthesis of Mo_1−*x*_W_*x*_S_2_ alloy and vertically composition-controlled Mo_1−*x*_W_*x*_S_2_ multilayer, which is promising as a photoactive material.

## Graphical abstract

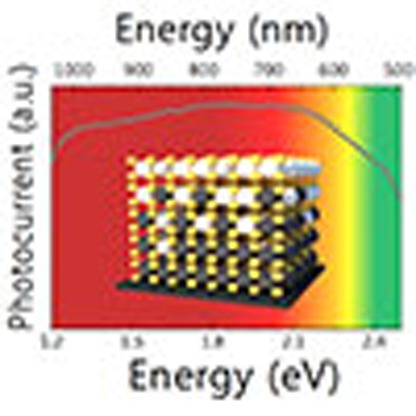

The band gap modulation of two-dimensional (2D) transition metal dichalcogenides (TMDCs) has been intensively studied, because of their various applications in optoelectronic devices such as photodiodes, phototransistors and solar cells^1^,^2^,^3^. It is well known that the band gap of 2D TMDCs is dependent on the number of layers^4^,^5^,^6^. In addition, alloying 2D TMDCs through the synthesis of Mo_1−*x*_W_*x*_S_2_, Mo_1−*x*_W_*x*_Se_2_ or MoS_2*x*_Se_2(1−*x*)_, for example, is another way of practically modulating the band gap. This is an effective approach because of the good thermodynamic stability at room temperature of the alloys, as predicted by theoretical calculations^7^,^8^,^9^,^10^,^11^,^12^,^13^,^14^. Furthermore, recent studies have shown that a vertically composition-controlled (VCC) 2D TMDCs multilayer is feasible for the high performance optoelectronic devices due to functionality of interlayer such as interlayer transition^15^,^16^,^17^,^18^,^19^,^20^,^21^,^22^. However, the reported synthesis processes for 2D TMDCs alloy and VCC 2D TMDCs multilayer, such as exfoliation, chemical vapor deposition and transfer, are limited in respect of systematic control of the composition and the number of layers, and clean interface for strong interlayer coupling^9^,^11^,^21^,^23^. Hence, an improved synthesis process for 2D TMDCs alloy and VCC 2D TMDCs multilayer is highly required.

Atomic layer deposition (ALD), which is based on surface reactions between precursors and reactants, has benefits such as high purity, thickness control on the atomic scale and large area uniformity^24^,^25^. In particular, it is suitable for the synthesis of alloy thin films with precisely controlled composition using the super-cycle method^26^,^27^,^28^. In addition, a continuous super-cycle process with different cycle ratios can produce a VCC multilayer with a clean interface^29^. In a previous report, we have shown that atomically thin, layer-controlled and wafer-level uniform 2D WS_2_ can be synthesized by sulfurization of ALD WO_3_ thin films^30^.

Here we report a synthesis method of Mo_1−*x*_W_*x*_S_2_ alloys by sulfurization of super-cycle ALD Mo_1−*x*_W_*x*_O_*y*_ alloy thin films. Using this method, we systematically control the composition and layer number (from mono- to tri-layers) of Mo_1−*x*_W_*x*_S_2_ alloys by controlling the cycle ratio between the ALD MoO_*x*_ and WO_3_. The bandgaps of the Mo_1−*x*_W_*x*_S_2_ alloys are precisely controlled as functions of the composition and layer numbers of each respective alloy, as measured based on the photoluminescence (PL) spectra. Scanning transmission electron microscopy (STEM) shows the mixing of Mo and W atoms with shared metal atom sites in monolayer Mo_1−*x*_W_*x*_S_2_ alloy. Furthermore, we develop a process to synthesize a VCC Mo_1−*x*_W_*x*_S_2_ multilayer using a sequential super-cycle ALD process—specifically, 5 continuous super-cycles of ALD with different cycle ratios for each super-cycle. Ultraviolet–visible spectrophotometer analysis shows that the synthesized VCC Mo_1−*x*_W_*x*_S_2_ multilayer has stronger interlayer coupling than that of a stacked VCC Mo_1−*x*_W_*x*_S_2_ multilayer fabricated by the individual transfer of each monolayer Mo_1−*x*_W_*x*_S_2_ alloy. This can be attributed to the clean interface between each layer in the synthesized sample^15^,^21^.

## Results

### MoS_2_ synthesis

**Figure 1:**
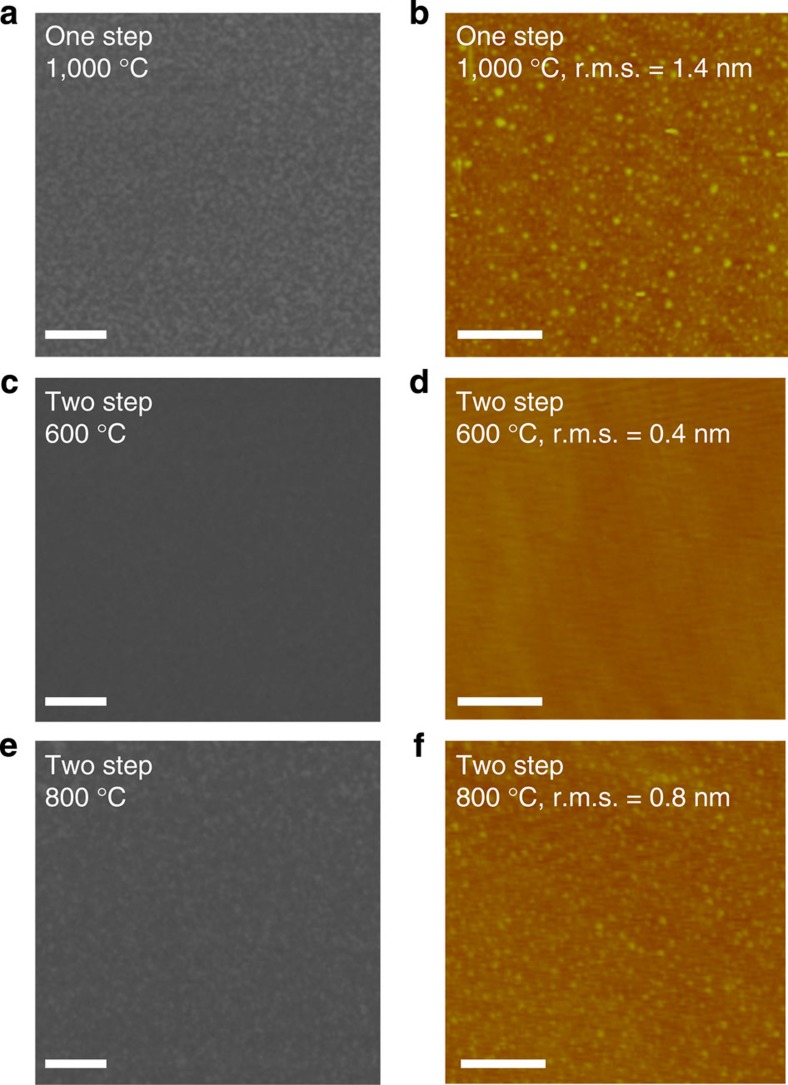
Sulfurization of MoO_*x*_ thin films. (**a**) SEM and (**b**) AFM images of sulfurized MoO_*x*_ thin film using one-step sulfurization process at 1,000 °C. SEM and AFM images of sulfurized MoO_*x*_ thin film using two-step sulfurization process with first-sulfurization temperatures of (**c**,**d**) 600 and (**e**,**f**) 800 °C, respectively. Scale bars, (**a**,**c**,**e**) 200 nm and (**b**,**d**,**f**) 0.5 μm.

Previously, we reported the synthesis of WS_2_ using sulfurization of ALD WO_3_ thin film with a one-step sulfurization process at 1,000 °C (ref. 30). These synthesized WS_2_ exhibit smooth and continuous surfaces with layer controllability from mono- to tetra-layer. Based on this result, we sulfurized ALD MoO_*x*_ thin film (nine cycles, optimization of ALD MoO_*x*_ is represented in Supplementary Fig. 1) using a one-step sulfurization process at 1,000 °C (see experimental section) to synthesize MoS_2_. Figure 1a,b comprises scanning electron microscope (SEM) and atomic force microscopy (AFM) images of sulfurized MoO_*x*_ thin film using the one-step sulfurization process at 1,000 °C. In contrast to WS_2_, however, the MoS_2_ shows a rough and non-continuous surface, and the measured root mean square (r.m.s.) is much larger (1.4 nm) than that of the SiO_2_ substrate (0.37 nm). We surmise that this discrepancy between the MoO_*x*_ and WO_3_ thin films sulfurized at the same temperature (1,000 °C) is caused by the relatively lower vaporization temperature of MoO_*x*_ (∼700 °C) in comparison with WO_3_ (over 1,100 °C)^31^. In other words, the MoO_*x*_ is vaporized before the conversion to MoS_2_ is complete, resulting in a rough surface.

Therefore, we examined the effect of sulfurization temperature on the roughness of the sulfurized ALD MoO_*x*_ thin film. To achieve this, we conducted a two-step sulfurization process, which consists of a low-temperature first step for the sulfurization of the MoO_*x*_ and a high-temperature second step to enhance the MoS_2_ crystallinity. The first-sulfurization temperatures were set to lower (600 °C) and higher (800 °C) temperatures than the vaporization temperature of MoO_*x*_ (700 °C), while the second-sulfurization temperature and process time were kept at 1,000 °C and 150 min, respectively (see Methods section). The roughness of the sulfurized MoO_*x*_ thin films in accordance with first-sulfurization temperature was then compared using SEM and AFM (Fig. 1c–f). Figure 1c,e shows SEM images of the sulfurized MoO_*x*_ thin films for first-sulfurization temperatures of 600 °C and 800 °C, respectively. The sulfurized MoO_*x*_ thin film at a first-sulfurization temperature of 600 °C has a smooth and continuous surface, while the MoO_*x*_ thin film sulfurized at a first-sulfurization temperature of 800 °C has a rough and non-continuous surface. AFM analyses (Fig. 1d,f) illustrate the variations in the roughness of the sulfurized MoO_*x*_ thin films more clearly, which is due to the differing first-sulfurization temperatures. The r.m.s. value for the MoS_2_ sulfurized at 600 °C is very low (∼0.4 nm) and is close to the r.m.s. value of the SiO_2_ substrate (0.37 nm). In contrast, the r.m.s. value of the MoS_2_ in the 800 °C case is relatively high (0.8 nm). As a result, a first-sulfurization temperature of 600 °C results in MoS_2_ with uniform and continuous surfaces, due to the fact that the first-sulfurization temperature is lower than the vaporization temperature of MoO_*x*_, as we assumed. Based on this result, we used a two-step sulfurization process with a 600 °C first-sulfurization temperature to synthesize continuous MoS_2_ and Mo_1−*x*_W_*x*_S_2_ alloys.

**Figure 2:**
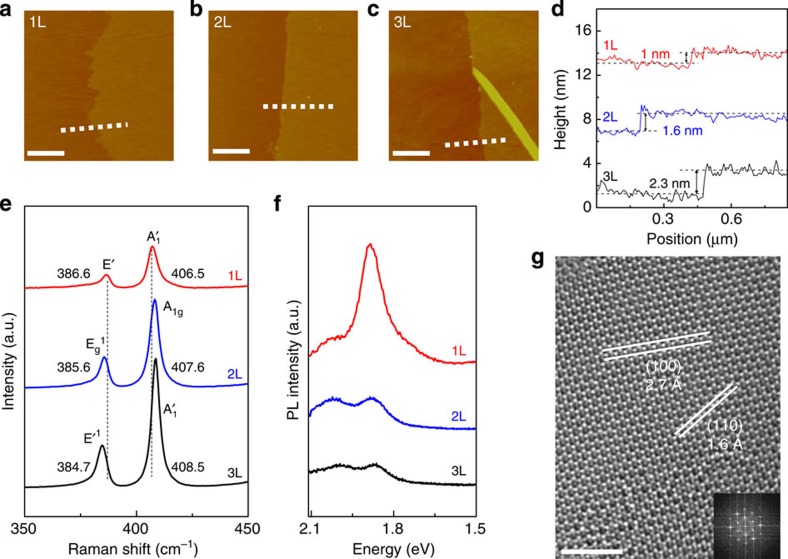
Characterization of MoS_2_. (**a**–**c**) AFM images and (**d**) height profiles (along with white dashed line in AFM images) of transferred MoS_2_ on SiO_2_ substrate for 1l, 2l and 3l thickness, respectively. Scale bars, 0.5 μm. (**e**) Raman spectra and (**f**) PL spectra for 1l (red), 2l (blue) and 3l (black) MoS_2_ on SiO_2_ substrate. (**g**) HRTEM image of 1l MoS_2_ at a selected region and (inset) FFT pattern. Scale bars, 2 nm.

Next, layer-number-controlled MoS_2_ was synthesized utilizing the two-step sulfurization process described above. Figure 2a–d shows the AFM images and height profiles of the transferred MoS_2_, which were synthesized by sulfurizing MoO_*x*_ thin films deposited by 6, 9 and 12 ALD cycles. The measured thicknesses of the synthesized MoS_2_ were ∼1, 1.6 and 2.3 nm for 6, 9 and 12 MoO_*x*_ ALD cycles, respectively. These thicknesses correspond to mono-, bi- and tri-layer (1, 2 and 3l) MoS_2_, considering that the height of 1l MoS_2_ on SiO_2_ is ∼1 nm and the spacing between the first and second MoS_2_ layers is ∼0.6 nm (refs 3, 4). As reported previously, the larger AFM-measured spacing between the first MoS_2_ layer and the substrate, compared with that between the MoS_2_ layers, is caused by the effect of distinct tip–sample and tip–substrate interactions^3^,^30^,^32^. Also, the apparent colour gains of the transferred 1, 2 and 3l MoS_2_ are observed in optical microscopy (OM) images (Supplementary Fig. 2). It should be noted that the MoS_2_ is not formed in the case of an ALD MoO_*x*_ thin film with an ALD cycle number of <3 (Supplementary Fig. 3). This is attributed to a nucleation delay during the initial growth of the MoO_*x*_, and similar behaviour was observed during the synthesis of WS_2_ by sulfurization of ALD WO_3_ (ref. 30). After the nucleation delay, 1l of MoS_2_ is formed by the sulfurization of each three-cycle ALD MoO_*x*_ thin film sample (∼0.8−0.9 nm in thickness). This observation agrees with a previous report, where ∼1 nm of MoO_*x*_ film transformed into a 1l MoS_2_ via sulfurization^33^. The stoichiometry calculated from X-ray photoemission spectroscopy (XPS) result is 2 (S/Mo) as shown in Supplementary Fig. 4. As a result, we can systematically control the layer number of MoS_2_ by controlling the ALD MoO_*x*_ cycle number.

The MoS_2_ were further characterized using Raman, PL and high-resolution TEM (HRTEM). The Raman spectra (*λ*_exc_=532 nm) for 1, 2l and 3l MoS_2_ are shown in Fig. 2e. The MoS_2_ exhibit in-plane and out-of-plane vibrations modes at 386.6 and 406.5 cm^−1^ (E′ and A′_1_) for the 1l, 385.6 and 407.6 cm^−1^ (E_g_^1^ and A_1g_) for 2l, and 384.7 and 408.5 cm^−1^ (E′^1^ and A′_1_) for 3l (ref. 34). From the Raman spectra, we calculated the relative peak distance between the in-plane and out-of-plane modes, which is closely related to the layer number of the MoS_2_ due to the softening in the in-plane and stiffening in the out-of-plane mode frequencies, with increasing layer numbers^35^,^36^. The calculated relative peak distances are 19.9, 22 and 23.8 cm^−1^ for the 1, 2 and 3l samples, respectively, which are in good agreement with previously reported values for synthesized MoS_2_ (refs 37, 38, 39).

The PL spectra dependence on the layer number of the MoS_2_ is shown in Fig. 2f. The spectrum of the 1l MoS_2_ shows PL peaks at 1.89 eV and 2.01 eV, which are correlated to the A_1_ and B_1_ direct excitonic transitions of the MoS_2_, respectively. With increasing layer number, weak PL peaks are observed at 1.87 eV and 2.00 eV for the 2l, and 1.86 eV and 1.99 eV for the 3l. The red shift and low intensity of the PL peaks with increasing layer number is due to the band gap transition from direct to indirect, which is consistent with the dependency of the PL peak on the layer number^4^,^5^,^6^,^40^. These Raman and PL results again confirm the layer controllability of MoS_2_ using the ALD process. Figure 2g is an HRTEM image for the synthesized 1l MoS_2_. The MoS_2_ shows a honeycomb-like structure with lattice spacing of 0.27 nm and 0.16 nm for the (100) and (110) planes, respectively. In addition, sixfold coordination symmetry is observed in the fast Fourier transformation (FFT) image (inset of Fig. 2g). The approximate domain size is 10−20 nm, similar to that of previously reported synthesized MoS_2_ and WS_2_ using the sulfurization of MoO_*x*_ and WO_3_ thin films^30^,^41^.

### Mo_1−*x*_W_*x*_S_2_ alloy synthesis

**Figure 3:**
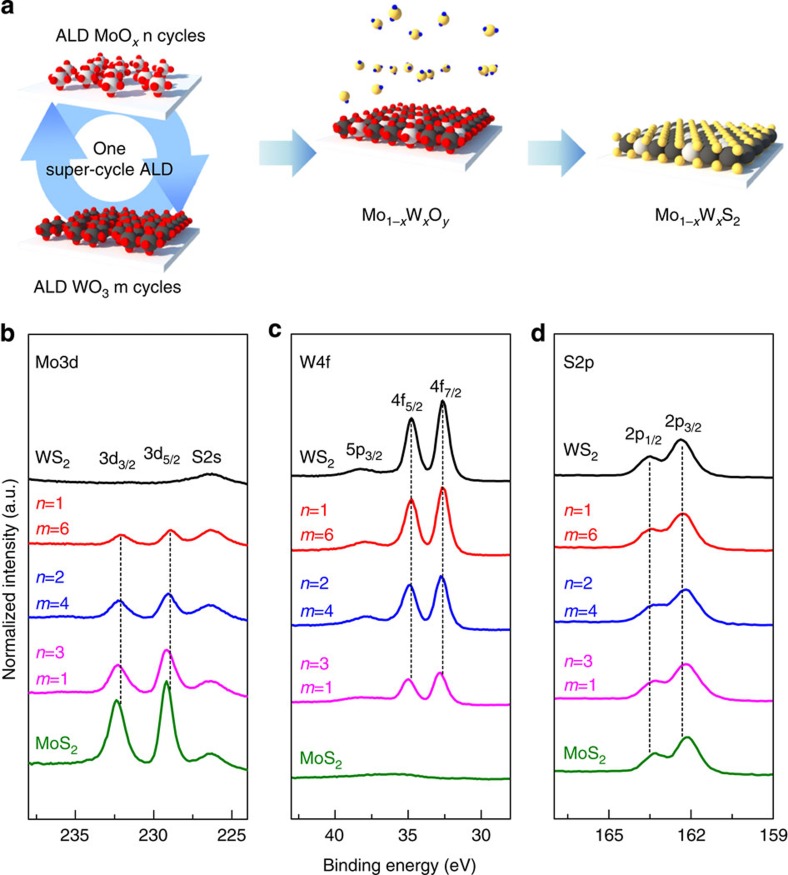
Synthesis and XPS of Mo_1−*x*_W_*x*_S_2_ alloy. (**a**) Synthesis procedure of super-cycle ALD for Mo_1−*x*_W_*x*_S_2_ alloy. XPS measurements for (**b**) Mo3d, (**c**) W4f and (**d**) S2p core levels in the 1l Mo_1−*x*_W_*x*_S_2_ alloy with different *n* and *m* numbers in one super-cycle. All measured XPS results are normalized by S2p_3/2_ peak intensity.

A super-cycle ALD-based Mo_1−*x*_W_*x*_S_2_ alloy synthesis process was developed based on the synthesis processes for 2D MoS_2_ (this study) and WS_2_ (previous study)^30^. The overall synthesis scheme for the Mo_1−*x*_W_*x*_S_2_ alloy is illustrated in Fig. 3a. First, we conducted 10 cycles of WO_3_ ALD to address the nucleation delay of the ALD WO_3_ (ref. 30) (not shown in Fig. 3a). Subsequently, one super-cycle ALD process consisting of *n* cycles of ALD MoO_*x*_ and *m* cycles of ALD WO_3_ was conducted and the deposited Mo_1−*x*_W_*x*_O_*y*_ alloy thin films were sulfurized. We used varying cycles for MoO_*x*_ (*n*) and WO_3_ (*m*) in one super-cycle to deposit 0.8−0.9-nm-thick composition-controlled Mo_1−*x*_W_*x*_O_*y*_ alloy thin films to create a 1l Mo_1−*x*_W_*x*_S_2_ alloy. This was based on the growth rate of ALD MoO_*x*_ (2.7 Å per cycle) and WO_3_ (0.9 Å per cycle), as shown in Supplementary Table 1. Figure 3b–d shows the XPS spectra of the 1l MoS_2_, 1l WS_2_ and sulfurized Mo_1−*x*_W_*x*_O_*y*_ alloy thin films with different *n* and *m* numbers in one super-cycle. All measured XPS results were normalized by S2p_3/2_ peak intensity and calibrated to the C1s peak at 285 eV. With increasing *n*/*m* ratio, the intensity of the Mo3d peaks increased, while the W5p_3/2_ and W4f peaks decreased. Furthermore, the peak positions for Mo3d and W4f gradually shifted to higher binding energies, from 232.2 eV and 229.1 eV to 232.5 eV and 229.4 eV for Mo3d_3/2_ and Mo3d_5/2_, respectively, and from 34.8 eV and 32.6 eV to 35.0 eV and 32.8 eV for W4f_5/2_ and W4f_7/2_, respectively. In addition, the S2p peaks shifted to lower binding energies, from 163.5 eV and 162.4 eV to 163.3 eV and 162.2 eV for S2p_1/2_ and S2p_3/2_, respectively. This small shift in peak position is attributed to the enhanced electron attraction strength of S and the reduced electron attraction strength of W, following increased Mo content due to smaller electronegativity of Mo (2.16) than that of W (2.36) as previously reported^7^. It is noteworthy that the Mo^6+^ 3d_3/2_ peak, which is attributed to the Mo–O bonding, is not observed in the Mo3d spectra; this indicates the absence of O species.

We calculated the Mo, W and S concentrations from the XPS results for the Mo3d, W4f and S2p peaks, respectively, to examine the Mo_1−*x*_W_*x*_S_2_ alloy composition. Table 1 presents the calculated concentration and W composition, *x*. The calculated *x* value is dependent on the *n* and *m* numbers in a single super-cycle, and yields *x*=0.8 for *n*=1 and *m*=6, *x*=0.6 for *n*=2 and *m*=4, and *x*=0.3 for *n*=3 and *m*=1. Also, the calculated stoichiometry is 2 (S/(Mo+W)). This shows that the W composition (*x*) in the Mo_1−*x*_W_*x*_S_2_ alloys can be systematically modulated by changing the values of *n* and *m* in one super-cycle.

**Figure 4:**
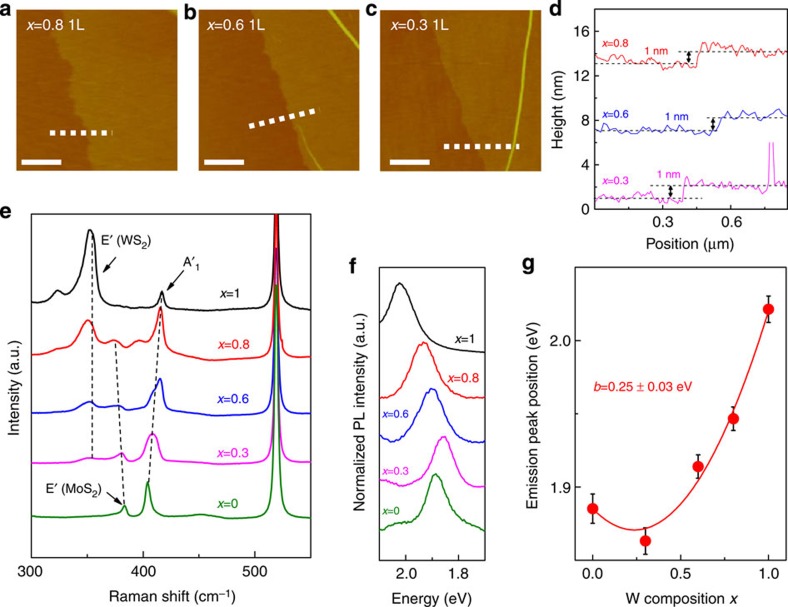
Characterization of Mo_1−*x*_W_*x*_S_2_ alloy. (**a**–**c**) AFM images and (**d**) height profiles (along with white dashed line in AFM images) of transferred 1l Mo_1−*x*_W_*x*_S_2_ alloy on SiO_2_ substrate for *x*=0.8, 0.6 and 0.3, respectively. Scale bars, 0.5 μm. (**e**) Raman spectra and (**f**) PL spectra for 1l Mo_1−*x*_W_*x*_S_2_ alloy on SiO_2_ substrate for *x*=1, 0.8, 0.6, 0.3 and 0. (**g**) PL peak position versus W composition (*x*) graph. Error bars represent s.d. of PL peak position in five-times repeatedly synthesized Mo_1−*x*_W_*x*_S_2_ alloy.

The synthesized composition-controlled Mo_1−*x*_W_*x*_S_2_ alloy from super-cycle ALD Mo_1−*x*_W_*x*_O_*y*_ alloy thin films were characterized using AFM, Raman and PL, as shown in Fig. 4. The AFM images and height profiles of the transferred Mo_1−*x*_W_*x*_S_2_ alloys are represented in Fig. 4a−d and they show good uniformity and continuity (also see OM images in Supplementary Fig. 5). The measured thicknesses of the Mo_0.2_W_0.8_S_2_, Mo_0.4_W_0.6_S_2_ and Mo_0.7_W_0.3_S_2_ alloys were all ∼1 nm, corresponding to the 1l thickness of Mo_1−*x*_W_*x*_S_2_ alloy. Furthermore, 2 and 3l Mo_1−*x*_W_*x*_S_2_ alloys can be synthesized using two- and three-super-cycle ALD Mo_1−*x*_W_*x*_O_*y*_ alloy thin films (Supplementary Fig. 6). As a result, our super-cycle ALD-based Mo_1−*x*_W_*x*_S_2_ alloy synthesis process can systematically control the layer number, as well as the composition of the resultant alloys through manipulation of the super-cycle ALD process.

Figure 4e shows the Raman spectra of composition-controlled 1l Mo_1−*x*_W_*x*_S_2_ alloys. The 1l WS_2_ (*x*=1) exhibits first-order modes: out-of-plane (A′_1_) and in-plane (E′) modes at 417 cm^−1^ and 357 cm^−1^, respectively, and a second-order mode: 2LA(M) at 353 cm^−1^ (ref. 30). The A′_1_ mode shifts to a lower frequency with decreasing W composition, while the E′ mode related to WS_2_ does not noticeably shift with the reduction of intensity. In addition, an E′ mode related to MoS_2_ appear at *x*=0.8 and shifted to a higher frequency with a reduction in W composition. The specific peak position dependency on W composition is represented in Supplementary Fig. 7, and the W composition dependence of the Raman spectra of the 1l Mo_1−*x*_W_*x*_S_2_ alloy is consistent with previous reports^7^,^8^,^9^.

The normalized PL spectra of the composition-controlled 1l Mo_1−*x*_W_*x*_S_2_ alloys are shown in Fig. 4f, also the *x* values versus the average PL peak positions and s.d. of five-times repeatedly synthesized 1l Mo_1−*x*_W_*x*_S_2_ alloys are plotted in Fig. 4g. As the value of *x* increased from 0 to 1, the averaged PL peak position initially decreases from 1.885 to 1.863 eV, and then gradually increases to 2.021 eV. This non-linear PL peak position behaviour with changing *x* is the so-called ‘bowing effect', and has also been reported for other semiconducting alloys and exfoliated 1l Mo_1−*x*_W_*x*_S_2_ alloys^9^,^42^,^43^. The bowing effect in 1l Mo_1−*x*_W_*x*_S_2_ alloy can be described by the Equation (1),


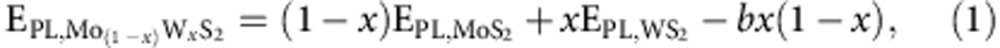


where *b* is a bowing parameter. After fitting the experimental results as shown in Fig. 4g (red solid curve), a *b* value of 0.25±0.03 eV was extracted. The extracted *b* value is comparable to that of the previous experiment (0.25±0.04 eV) and simulation (0.28±0.04 eV) results^9^. Furthermore, the s.d. of the five-times repeatedly synthesized 1l Mo_1−*x*_W_*x*_S_2_ alloys are small within the range of 0.008 to 0.01, which indicates that the process has good reliability in terms of composition control. Thus, the PL result confirms that we modulate the band gap of the Mo_1−*x*_W_*x*_S_2_ alloy by reliably controlling the composition. Moreover, the band gap can also be modulated by controlling the layer number (see Supplementary Fig. 8).

**Figure 5:**
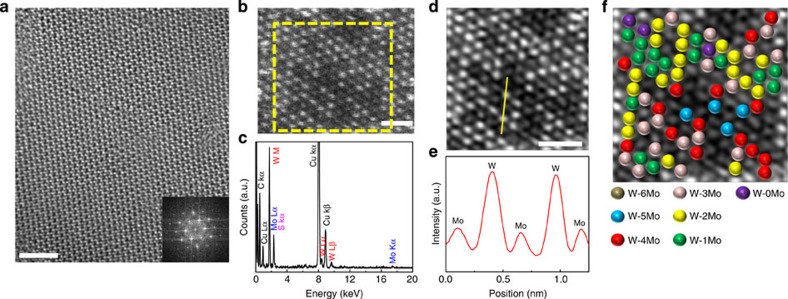
Atomic arrangement and mixture of Mo_0.4_W_0.6_S_2_ alloy. (**a**) HRTEM image of 1l Mo_0.4_W_0.6_S_2_ alloy at a selected region, and (inset) FFT pattern. Scale bars, 2 nm. (**b**) STEM-ADF image of 1l Mo_0.4_W_0.6_S_2_ alloy at a selected region and (**c**) corresponding EDX spectrum. Scale bars, 1 nm. (**d**) Inverse FFT image with masking applied to yellow dashed square region in **b**. Scale bars, 1 nm. (**e**) Intensity profile of yellow solid line in **d**. (**f**) Coloured W atoms with light brown, blue, red, dark red, yellow, green and violet for six, five, four, three, two, one and zero number of neighbouring Mo atoms.

Figure 5a is the HRTEM image of the 1l Mo_0.4_W_0.6_S_2_ alloy (*x*=0.6). The Mo_0.4_W_0.6_S_2_ alloy shows a periodic atomic arrangement with a honeycomb-like structure and sixfold coordination symmetry, similar to the 1l MoS_2_ shown in Fig. 2f. To distinguish between the W and Mo atoms in the 1l Mo_0.4_W_0.6_S_2_ alloy, we analysed the Mo_0.4_W_0.6_S_2_ alloy using STEM annular dark-field and energy dispersive X-ray spectrometry (EDX). Figure 5b is the STEM-ADF image of the 1l Mo_0.4_W_0.6_S_2_ alloy. Brighter and less bright spots, which correspond to W and Mo atoms, respectively, are clearly resolved in the ADF image, as previously reported^44^. The calculated Mo/W ratio from the atom count in Fig. 5b is 0.42:0.58, which differs by <5% from the XPS-measured stoichiometry. In addition, the EDX result in Fig. 5c supports the presence of W, Mo and S species in the 1l Mo_0.4_W_0.6_S_2_ alloy. To extract a clear intensity difference between the W and Mo atoms, we performed an inverse FFT by applying a mask to the yellow dashed square region in Fig. 5b. Figure 5d–e shows the inversed FFT image (Fig. 5d) and intensity profile (Fig. 5e) along with the yellow solid line in Fig. 5d. Although S atoms are not distinguishable in our result as a result of the displacement of S atoms at 200 kV operation voltage by the knock-on mechanism^45^, the W and Mo atoms are clearly observable, confirming that these elements share the metal atom sites^44^. The preference for Mo or W atoms at the neighbouring sites of W atoms is evaluated by degree of alloying that can be calculated by Equation (2)^23^,^44^,


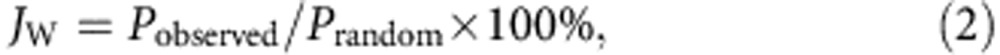


where *P*_observed_ is the averaged ratio of number of neighbouring Mo atoms to total neighbouring sites of W atoms, and *P*_random_ is the total ratio of Mo atoms in the examined layer. Figure 5f represented differently coloured W atoms depending on number of neighbouring Mo atoms: light brown, blue, red, dark red, yellow, green and violet for six, five, four, three, two, one and zero number of neighbouring Mo atoms. The calculated degree of alloying is 99%, which indicate that there is no preference for Mo or W atoms at the neighbouring sites of W atoms and a random mixture of our 1l Mo_1−*x*_W_*x*_S_2_ alloy.

### A VCC Mo_1−*x*_W_*x*_S_2_ synthesis

**Figure 6:**
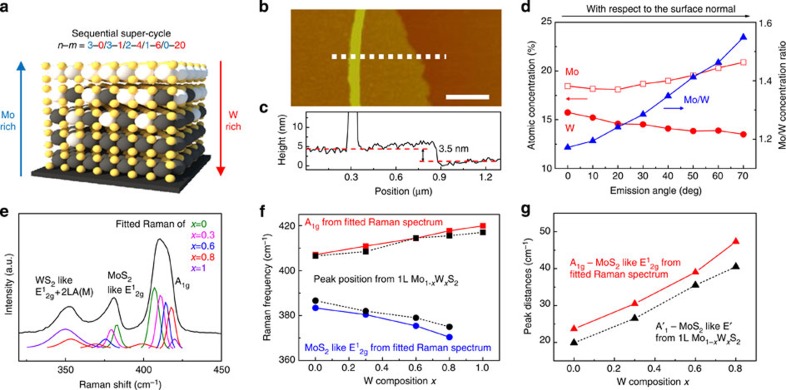
Characterization of VCC Mo_1−*x*_W_*x*_S_2_ multilayer. (**a**) Sequential super-cycle ALD procedure and schematic structure of a VCC Mo_1−*x*_W_*x*_S_2_ multilayer. (**b**) AFM image and (**c**) height profiles (along with white dashed line in AFM image) for a VCC Mo_1−*x*_W_*x*_S_2_ multilayer. Scale bars, 0.5 μm. (**d**) Calculated atomic concentration and relative concentration ratio of Mo and W from ARXPS measurement. (**e**) Raman spectra for a VCC Mo_1−*x*_W_*x*_S_2_ multilayer. (**f**) Raman peak position of A_1g_ and MoS_2_-like E^1^_2g_ modes from fitted Raman spectra (red and blue solid line) and from measured Raman spectra of 1l Mo_1−*x*_W_*x*_S_2_ alloy (black dashed line). (**g**) Calculated Raman peak distances between A_1g_ and MoS_2_-like E^1^_2g_ modes from fitted Raman spectra (red solid line) and from measured Raman spectra of 1l Mo_1−*x*_W_*x*_S_2_ alloy (black dashed line).

The composition controllability of our ALD-based Mo_1−*x*_W_*x*_S_2_ alloy synthesis process enables synthesis of a VCC Mo_1−*x*_W_*x*_S_2_ multilayer with a clean interface, strong interlayer coupling and broadband light absorption. We sulfurized a VCC Mo_1−*x*_W_*x*_O_*y*_ thin film that was deposited by a sequential super-cycle ALD process, so as to synthesize a VCC Mo_1−*x*_W_*x*_S_2_ multilayer, as shown in Fig. 6a. First, we conducted 20 cycles of WO_3_ ALD on a SiO_2_ substrate, corresponding to 1l WS_2_. We immediately performed three super-cycles of Mo_1−*x*_W_*x*_O_*y*_ ALD with different super-cycle *n* and *m* numbers, in the following order: *n*=1 and *m*=6, *n*=2 and *m*=4, and *n*=3 and *m*=1. Last, we conducted three cycles of MoO_*x*_ ALD (*n*=3) corresponding to 1l MoS_2_. The deposited VCC Mo_1−*x*_W_*x*_O_*y*_ thin film was sulfurized to convert it into a VCC Mo_1−*x*_W_*x*_S_2_ multilayer. Figure 6b,c shows an AFM image and height profile of the transferred VCC Mo_1−*x*_W_*x*_S_2_ multilayer, with a measured thickness of ∼3.5 nm. This thickness, synthesized by five sequential ALD super-cycles, corresponds to a 5l Mo_1−*x*_W_*x*_S_2_ alloy, which is consistent with each super-cycle result for the 1l Mo_1−*x*_W_*x*_S_2_ alloy.

The different composition concentrations of the bottom and top layers in the VCC Mo_1−*x*_W_*x*_S_2_ multilayer were analysed using angle-resolved XPS (ARXPS). Figure 6d shows the calculated atomic and relative concentration ratios of the Mo and W from the ARXPS measurement (ARXPS spectra are shown in Supplementary Fig. 9). The Mo concentration increased from 18.6 to 20.9%, while the W concentration decreased from 15.7 to 13.5%, with increasing emission angle from 0 to 70° (red line). The Mo/W concentration ratio increased from 1.17 to 1.55 with increasing emission angle (blue line). Although the exact atomic concentration according to position in the VCC Mo_1−*x*_W_*x*_S_2_ multilayer cannot be calculated because of the larger depth resolution of the XPS measurement in comparison with the VCC Mo_1−*x*_W_*x*_S_2_ multilayer thickness, the emission angle dependency of the Mo and W concentration indicates Mo-rich and W-rich concentration in the upper and lower layers of the VCC Mo_1−*x*_W_*x*_S_2_ multilayer, respectively. As a result, ARXPS shows that the VCC Mo_1−*x*_W_*x*_S_2_ multilayer has VCC characteristics. Notably, the calculated stoichiometry ratio was 2 (S/(Mo+W)) in all ARXPS results.

The formation of Mo_1−*x*_W_*x*_S_2_ alloy with different compositions in a VCC Mo_1−*x*_W_*x*_S_2_ multilayer was analysed using Raman spectroscopy. Figure 6e shows the Raman spectrum of a VCC Mo_1−*x*_W_*x*_S_2_ multilayer, which exhibits strong peaks for A_1g_, MoS_2_-like E^1^_2g_ and WS_2_-like E^1^_2g_+2LA(M) modes. Each Raman peak can be fitted using a Lorentzian function to the Raman spectrum of the Mo_1−*x*_W_*x*_S_2_ alloy with *x*=0, 0.3, 0.6, 0.8 and 1. The fitted Raman spectrum was compared with the measured Raman spectrum for the 1l Mo_1−*x*_W_*x*_S_2_ alloy, with respect to variations in the peak position and peak distances of the A_1g_ and MoS_2_-like E^1^_2g_ modes, depending on W concentration. The A_1g_ and MoS_2_-like E^1^_2g_ peak positions from the fitted Raman spectrum are represented in Fig. 6f with the measured Raman peak positions for the 1l Mo_1−*x*_W_*x*_S_2_ alloy (black dashed line, the same as Supplementary Fig. 7). The variation in the fitted Raman peak position with increasing W concentration in the Mo_1−*x*_W_*x*_S_2_ alloy is the same as the variation in the measured Raman peak position for the 1l Mo_1−*x*_W_*x*_S_2_ alloy: A_1g_ shifts to a higher frequency with an increase in W concentration, while the MoS_2_-like E^1^_2g_ modes downshift. Figure 6g shows peak distances between the A_1g_ and MoS_2_-like E^1^_2g_ modes from the fitted Raman spectrum (red solid line) and measured Raman spectrum of the 1l Mo_1−*x*_W_*x*_S_2_ alloy (black dashed line), which are 3−4 cm^−1^ larger than that of the Raman spectrum of the 1l Mo_1−*x*_W_*x*_S_2_ alloy. This is due to the softening in the MoS_2_-like E^1^_2g_ mode frequency and stiffening in the A_1g_ mode frequency. Similar behaviour, that is, increasing peak distances with increasing layer number, is also observed in MoS_2_ (refs 35, 36) and WS_2_ (ref. 30) because of the reduced long-range Coulomb interaction between the effective charges, which is induced by an increase in the dielectric screening. These results for the fitted Raman spectra are in good agreement with the dependency of the peak positions on the W composition given by the measured Raman results, and the dependency of the peak distances on layer number in 2D TMDCs. Thus, we can conclude that the fitted Raman spectra show the formation of a Mo_1−*x*_W_*x*_S_2_ alloy with different compositions in a VCC Mo_1−*x*_W_*x*_S_2_ multilayer.

As a result, it can be stated that the ARXPS and Raman results show the VCC characteristics of a VCC Mo_1−*x*_W_*x*_S_2_ multilayer. Also, these findings indicate that the vertical interdiffusion of the Mo and W atoms during the sulfurization process have no critically effect on the VCC characteristics. A similar result was observed in a previous report, in that MoO_*x*_/WO_3_ thin film was converted to MoS_2_/WS_2_ without the formation of a Mo_1−*x*_W_*x*_S_2_ alloy, indicating the limited interdiffusion of Mo and W atoms^46^. Further, it is noteworthy that we verified the validity of ARXPS and Raman measurements as a means of characterizing the VCC Mo_1−*x*_W_*x*_S_2_ multilayer via characterization of a VCC Mo_1−*x*_W_*x*_S_2_ multilayer synthesized with a reversed vertical composition profile (see Supplementary Fig. 10).

**Figure 7:**
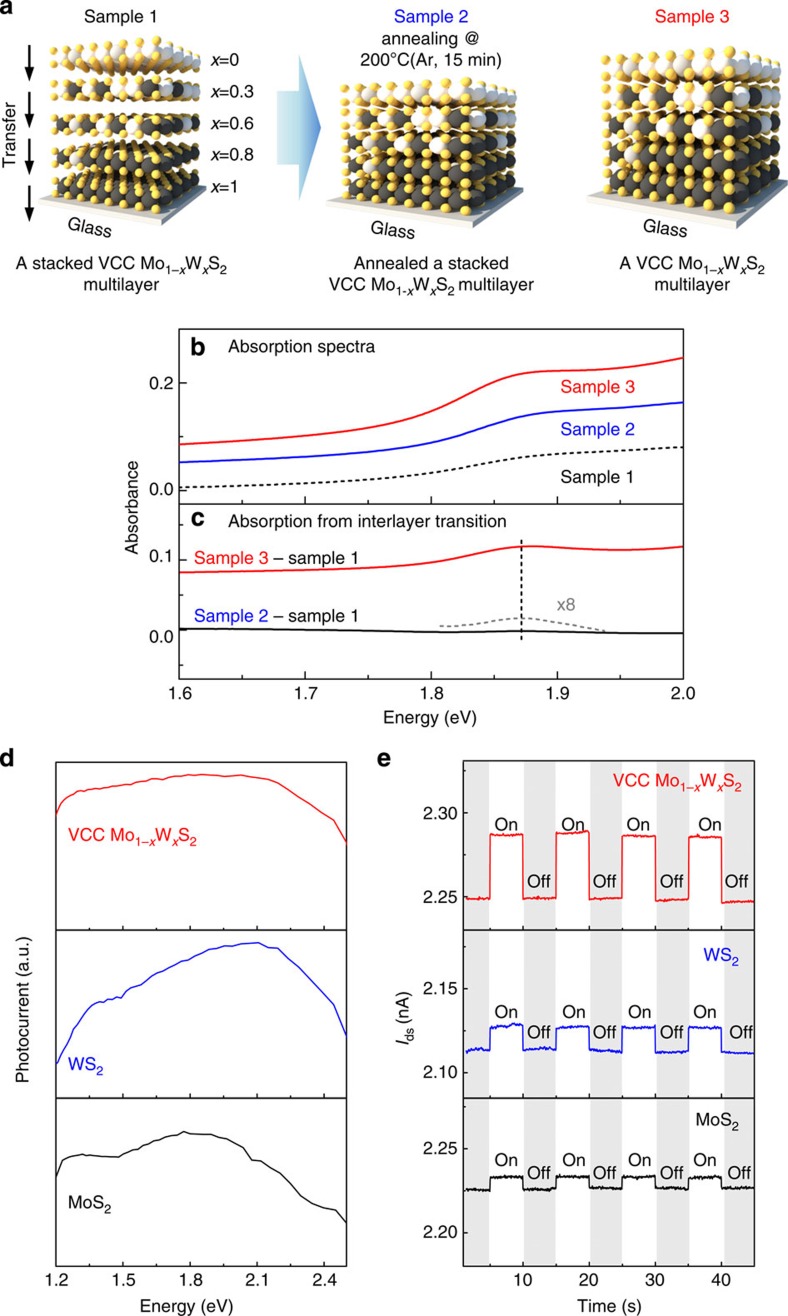
Absorbance and photoinduced current of VCC Mo_1−*x*_W_*x*_S_2_ multilayer. (**a**) Schematics of three sample types for ultraviolet–visible spectrophotometer measurement. (**b**) Absorption spectra of sample 1 (black solid line), sample 2 (blue solid line), and sample 3 (red solid line) and (**c**) extracted absorption spectra of interlayer transition using subtraction of sample 1 from sample 2 (black solid line) and from sample 3 (red solid line). (**d**) Spectral and (**e**) time-resolved photocurrent of a VCC Mo_1−*x*_W_*x*_S_2_ multilayer, 5l WS_2_ and 5l MoS_2_ photodetectors.

Since the interlayer coupling affects interlayer transition^15^,^21^,^47^,^48^, strong interlayer coupling in a synthesized VCC Mo_1−*x*_W_*x*_S_2_ multilayer was evaluated using comparison of interlayer transition in three difference sample types as shown in Fig. 7a. Sample 1 is a stacked VCC Mo_1−*x*_W_*x*_S_2_ multilayer fabricated by the transfer of each differently composed Mo_1−*x*_W_*x*_S_2_ alloy onto glass substrate, while sample 2 is the same as sample 1 but annealed at 200 °C for 15 min in an Ar ambient atmosphere to enhance the interlayer coupling by the removal of residual molecules^21^,^48^. Sample 3 is a transferred VCC Mo_1−*x*_W_*x*_S_2_ multilayer on glass substrate, which was annealed at 200 °C for 15 min in an Ar ambient atmosphere. Ultraviolet–visible spectrophotometer measurements for samples 1, 2 and 3 (Fig. 7b) illustrate that these have broadband light absorption properties due to the sum of the light absorption from the differently composed Mo_1−*x*_W_*x*_S_2_ alloys. In previous reports, the absorption spectrum of the interlayer transition could be obtained by comparing the intensity difference between the absorption spectra of the weakly interlayer-coupled sample and that of the strongly interlayer-coupled sample^15^,^48^. Based on these reports, we extracted the interlayer transition absorption spectrum by subtracting the absorption spectrum of sample 1 from that of sample 2 and of sample 3, since sample 1 has the weakest interlayer coupling of the three samples as a result of the contamination at the interface caused by the layer transfer process^15^,^21^,^48^. The extracted absorption spectra of the interlayer transition are shown in Fig. 7c. The sample 2–sample 1 spectrum (black solid line) shows a small absorbance peak at 1.87 eV, while the sample 3–sample 1 spectrum (red solid line) shows an absorbance peak that is over five times stronger than the sample 2–sample 1 absorbance peak at the same position. Specific observations on the origin of the absorbance peak position from the interlayer transition (1.87 eV) are described in the Supplementary Information (Supplementary Fig. 11). The stronger absorbance peak of sample 3–sample 1 in comparison with that of sample 2–sample 1 indicates that sample 3 has stronger interlayer coupling compared with sample 2. In other words, a VCC Mo_1−*x*_W_*x*_S_2_ multilayer based on sequential super-cycle ALD has the strongest interlayer coupling among the three types of samples. We surmise that this strong interlayer coupling results from the absence of a transfer process, which eliminates the incorporation of residual molecules such as H_2_O and organic contaminants^15^,^21^,^47^,^48^.

A VCC Mo_1−*x*_W_*x*_S_2_ multilayer exhibits a broadband light absorption property, as well as strong interlayer coupling. Thus, the VCC Mo_1−*x*_W_*x*_S_2_ multilayer has promising potential use as an active layer in an efficient photodetector. To evaluate the photoinduced response of the VCC Mo_1−*x*_W_*x*_S_2_ multilayer, we observed the spectral and time-resolved photocurrent of a VCC Mo_1−*x*_W_*x*_S_2_ multilayer photodetector and compared it with 5l WS_2_ and 5l MoS_2_ photodetectors (see the Methods section for details of device fabrication, and see Supplementary Fig. 12 for AFM images of the 5l MoS_2_ and WS_2_ and their *I*–*V* characteristics). Figure 7d shows the dependence of the photocurrent on the illumination energy for the VCC Mo_1−*x*_W_*x*_S_2_ multilayer, 5l WS_2_, and 5l MoS_2_ photodetectors for a voltage drain to source (*V*_ds_) of 5 V. The continuum power spectral density is represented in Supplementary Fig. 13. The VCC Mo_1−*x*_W_*x*_S_2_ multilayer photodetector generates a broadband photoinduced current from 1.2 to 2.5 eV, because of its broadband light absorption property. In contrast, the 5l WS_2_ and 5l MoS_2_ photodetectors generate narrower photocurrents than the VCC Mo_1−*x*_W_*x*_S_2_ multilayer, at 1.3 and 2.1 eV for the 5l WS_2_ photodetector and 1.2 and 1.8 eV for the MoS_2_ device; these values correspond to the 5l WS_2_ and MoS_2_ bandgaps. We then examined the time-resolved photocurrent measurement using white-light illumination, as shown in Fig. 7e (result using specific laser wavelength is shown in Supplementary Fig. 14). The white light was first turned off for a period of 5 s, and then turned on for 5 s with the biasing *V*_ds_=5 V. The drain current (*I*_ds_) increased on activation of the light and decayed following removal of the incident light. The induced photocurrents were 39 pA, 13 pA and 11 pA for the VCC Mo_1−*x*_W_*x*_S_2_ multilayer, 5l WS_2_ and 5l MoS_2_ devices, respectively. Hence, the VCC Mo_1−*x*_W_*x*_S_2_ multilayer generates three to four times greater photocurrent than 5l WS_2_ or 5l MoS_2_, which is attributed to broadband light absorption. Thus, we concluded that the VCC Mo_1−*x*_W_*x*_S_2_ multilayer is promising as regards use as an efficient photodetector with broadband light absorption. Furthermore, the broadband light absorption property is feasible for various optoelectronic applications such as solar cells^49^,^50^.

## Discussion

In summary, we developed an ALD-based Mo_1−*x*_W_*x*_S_2_ synthesis process using sulfurization of super-cycle ALD Mo_1−*x*_W_*x*_O_*y*_ thin film. We studied the sulfurization process of ALD MoO_*x*_ thin films to produce uniform and continuous MoS_2_. The synthesized ALD-based Mo_1−*x*_W_*x*_S_2_ alloy show good stoichiometry, uniform and continuous surfaces, controlled composition and layer numbers, and mixing of Mo and W atoms. Moreover, we developed a simple method to synthesize a VCC Mo_1−*x*_W_*x*_S_2_ multilayer with a clean interface, which shows stronger interlayer coupling than that of a stacked VCC Mo_1−*x*_W_*x*_S_2_ multilayer fabricated using the transfer process. Further, we have shown that the VCC Mo_1−*x*_W_*x*_S_2_ multilayer has promising potential applications as an efficient photodetector, because of its broadband light absorption capability. It should also be noted that the ALD-based TMDCs alloy synthesis process is not only limited to Mo_1−*x*_W_*x*_S_2_, and we expect that similar process strategies can be developed for other TMDCs materials and their vertical stacks.

## Methods

### MoO_*x*_ film growth and characteristics

A 6-inch ALD chamber containing a loadlock chamber was used for the deposition of the MoO_*x*_ films. The films were deposited on SiO_2_(300 nm)/Si substrates by plasma-enhanced ALD using Mo(CO)_6_ and O_2_ plasma at a 200 °C growth temperature. The temperature of the bubbler containing Mo(CO)_6_ was maintained at 35 °C to produce adequate vapour pressure, and vapourized Mo(CO)_6_ molecules were transported into the chamber by pure argon (99.999%) carrier gas. The O_2_ flow and plasma power were fixed at 300 s.c.c.m. and 200 W, respectively. An ALD cycle consists of four steps: Mo(CO)_6_ precursor exposure (*t*_s_), Ar purging (*t*_p_), O_2_ plasma reactant exposure (*t*_r_) and another Ar purging (*t*_p_). In the ALD MoO_*x*_ process, the *t*_s_, *t*_p_ and *t*_r_ were fixed at 5 s, 12 s and 5 s, respectively. Optimization of the ALD MoO_*x*_ process is described in the Supplementary Fig. 1.

### Mo_1−*x*_W_*x*_O_*y*_ Film Growth

MoO_*x*_ and WO_3_ ALD processes^27^ were used to deposit Mo_1−*x*_W_*x*_O_*y*_ film using super-cycle ALD (as shown in Fig. 3a) under the same chamber and deposition conditions described above for the ALD of MoO_*x*_. After 10 cycles of WO_3_ ALD to address nucleation delay^27^, we conducted super-cycle ALD, which consists of *n* cycles of MoO_*x*_ ALD and *m* cycles of WO_3_ ALD. The detailed process steps are shown in Supplementary Table 1.

### Sulfurization processes

*One-step process*. To sulfurize the ALD MoO_*x*_, the sample was placed in the centre of a tube furnace (1.2 inch in diameter). Initially, the sample was heated at 200 °C for 60 min under flowing H_2_ (25 s.c.c.m.) and Ar (25 s.c.c.m.) gas, to remove any organic contaminants on the surface. Subsequently, the temperature was gradually increased from 200 to 1,000 °C at 13.3 °C min^−1^, and this temperature was then maintained for 60 min with flowing Ar (50 s.c.c.m.) and H_2_S (5 s.c.c.m.). Then, the sample was cooled to room temperature under a flowing Ar (50 s.c.c.m.) atmosphere.

*Two-step process*. Initially, samples were annealed at 200 °C as in the one-step process. Then, the temperature was gradually increased from 200 °C to first-sulfurization temperatures of 600 or 800 °C at 13.3 °C min^−1^. The peak temperature (600 or 800 °C) was maintained for 60 min with flowing Ar (50 s.c.c.m.) and H_2_S (5 s.c.c.m.). Subsequently, the temperature was gradually increased from the first-sulfurization temperatures to 1,000 °C at 13.3 °C min^−1^ and was maintained at this temperature for 30 min with flowing Ar (50 s.c.c.m.) and H_2_S (5 s.c.c.m.). The sample was cooled to room temperature under a flowing Ar (50 s.c.c.m.) atmosphere after the process was completed. Based on the results shown in Fig. 1, we used a two-step sulfurization process with a 600 °C first-sulfurization temperature to sulfurize MoO_*x*_ and Mo_1−*x*_W_*x*_O_*y*_ thin films in the MoS_2_ and Mo_1−*x*_W_*x*_S_2_ alloy synthesis process.

### Transfer of MoS_2_ and Mo_1−*x*_W_*x*_S_2_

The as-synthesized MoS_2_ and Mo_1−*x*_W_*x*_S_2_ on the SiO_2_ substrate were coated with polymethyl methacrylate (PMMA) by spin coating at 4,000 r.p.m. for 60 s. After curing of the PMMA at 100 °C for 15 min, the samples were immersed in 10% hydrogen fluoride solution to etch the SiO_2_ layer. Subsequently, the samples were washed using deionized (DI) water and scooped onto a clean SiO_2_/Si substrate. The PMMA was removed by acetone and washed away using isopropyl alcohol.

### Characterization of MoS_2_ and Mo_1−*x*_W_*x*_S_2_

OM (Olympus DX51), Raman spectroscopy (HORIBA, Lab Ram ARAMIS; 532-nm laser excitation wavelength), AFM (VEECO, Multimode), PL (SPEX1403, SPEX; 532-nm laser excitation wavelength), absorbance with ultraviolet–visible spectrophotometer (JASCO Corporation, V-650), XPS (Thermo UK, K-alpha), SEM (JEOL Ltd, JSM-6701F), TEM (FEI Titan G2 Cube 60-300; accelerating voltage, 80 kV), STEM and EDX (JEM 2100F; accelerating voltage, 200 kV) analyses were employed to characterize the MoS_2_ and Mo_1−*x*_W_*x*_S_2_ alloy, and a VCC Mo_1−*x*_W_*x*_S_2_ multilayer.

### Fabrication and characterization of photodetectors

Photodetectors were fabricated from an as-synthesized VCC Mo_1−*x*_W_*x*_S_2_ multilayer, 5l WS_2_ and 5l MoS_2_ on a SiO_2_ (300 nm)/Si substrate by evaporating Au(40 nm)/Ti(1 nm) electrodes with 100-μm channel length. Electrical measurements were conducted using a Keithley 2400 (Keithley Instruments). The photocurrent was measured by modulating the laser beam with a mechanical chopper (1,000 Hz) and detecting the photocurrent with a current preamplifier and a lock-in amplifier. A monochromator was used for wavelength-dependent measurements of the photocurrent.

### Parameters for analysis of XPS and Raman

We used Spectral Data Processor v4.1 for the XPS and Raman spectra fitting. In the fitting analysis of the XPS spectra, the full widths at half maximum (FWHM) were between 1.7 and 1.9 eV, the Lorentzian Gaussian Ratio was 2:8, the energy difference between the Mo3d spin-orbit doublet was set to 3.2 eV and the branching ratio was 2/3. In addition, we used Scofield Relative Sensitivity Factor for calculation of stoichiometry as represented in Supplementary Table 2. For the Raman spectrum fitting analysis, the FWHM was between 7 and 12 cm^−1^.

## Additional information

**How to cite this article:** Song, J. G. *et al*. Controllable synthesis of molybdenum tungsten disulfide alloy for vertically composition-controlled multilayer. *Nat. Commun.* 6:7817 doi: 10.1038/ncomms8817 (2015).

## Supplementary Material

Supplementary Information Supplementary Figures 1-14, Supplementary Tables 1-2 and Supplementary References

## Figures and Tables

**Table 1:** Calculated composition of Mo_1−*x*_W_*x*_S_2_.

**One super-cycle**		**Mo (%)**	**W (%)**	**S (%)**	**W composition x**
***n***	***m***				
1	6	6.9	26.5	66.6	0.8
2	4	12.5	19.3	68.2	0.6
3	1	22.5	10.5	67	0.3

Quantification analysis for Mo3d, W4f and S2p peaks depending on *n* and *m* number in one super-cycle. The calculation error for the composition is under 1%.
