# Supplementary material for: Controllable synthesis of molybdenum tungsten disulfide alloy for vertically composition-controlled multilayer
Source: Nat Commun. 2015 Jul 23;6:7817. doi: 10.1038/ncomms8817 (PMC4525162; doi:10.1038/ncomms8817)
Supplement: Supplementary Information — Supplementary Figures 1-14, Supplementary Tables 1-2 and Supplementary References [file ncomms8817-s1.pdf]

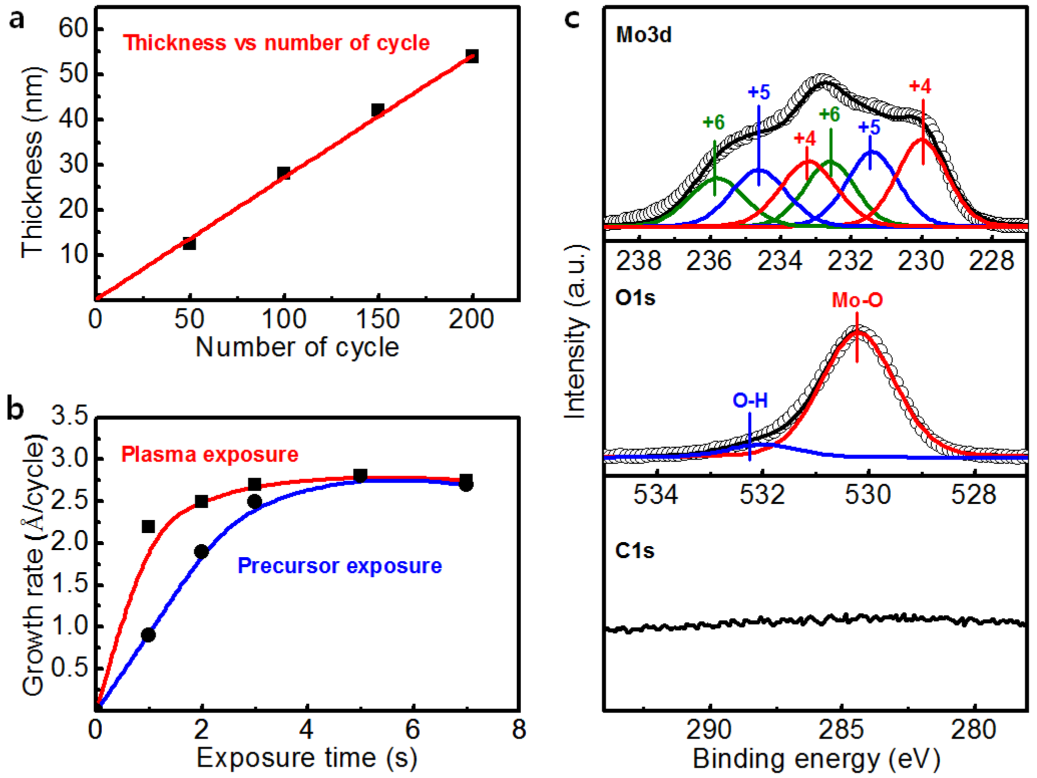

Supplementary Figure 1. (**a**) MoO_x thickness with increasing ALD cycle numbers. From the linear fit of the plots, the growth rate was determined to be 2.7 Å/cycle, and the growth is almost linear from the initial growth. The linearity of the graph indicates that the MoO_x-film thickness can be precisely controlled by adjusting the number of ALD cycles. (**b**) Growth rate according to increasing plasma and precursor exposure times. The saturation of the growth rate is observed after 5 and 3 s respectively, indicating that the ALD has a self-limiting growth characteristic. (**c**) XPS spectrum of Mo3d, O1s, and C1s core levels in ALD MoO_x film. The Mo3d results can be deconvoluted to form six peaks, which correspond to the three states: $Mo3d^{+6}$, $Mo3d^{+5}$, and $Mo3d^{+4}$. There are two peaks for each group: $Mo3d_{3/2}$ (higher energy) and $Mo3d_{5/2}$ (lower energy). A peak corresponding to O1s can be observed at 530.2 eV. The stoichiometric ratio, O/Mo, was 2.1 and no carbon species were detected in the C1s core level. As a result, we obtained high-purity MoO_x thin film using the ALD process.

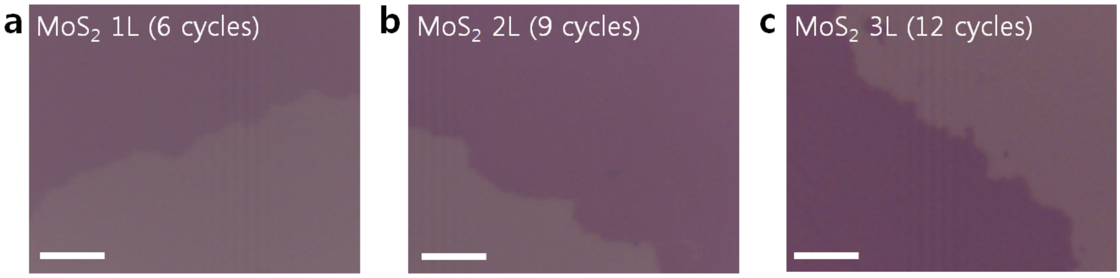

Supplementary Figure 2. Optical microscopy images of (**a**, **b** and **c**) 1L, 2L, and 3L MoS_2 after transference onto SiO_2 (285 nm) substrate. The numbers in parentheses refer to the MoO_x ALD cycle number. Scale bars, 5 µm.

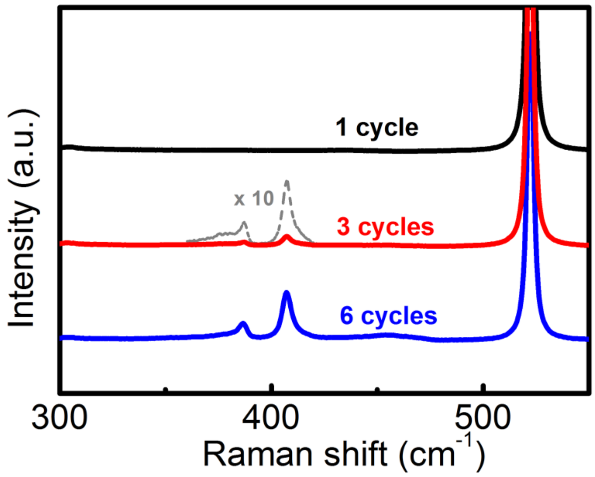

Supplementary Figure 3. The ALD-based MoS_2 were synthesized with 1, 3, and 6 cycles of MoO_x ALD on SiO_2 (285 nm) substrates and analyzed using Raman spectroscopy. No Raman peak is observed for the 1–cycle sample, while very weak Raman peaks appeared for the 3–cycle sample, i.e., E^1_{2g} and A_{1g} modes, indicating the presence of a MoS_2. Strong Raman peaks were observed after 6 cycles, which implies nucleation delay in the ALD MoO_x, similar to the case of the ALD-based WS_2[1].

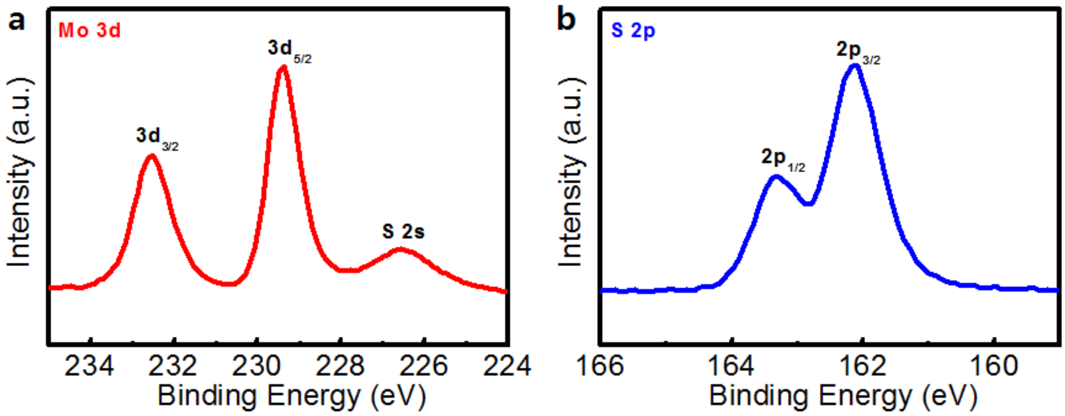

Supplementary Figure 4. The XPS spectra of the 1L MoS_2 at the (**a**) Mo3d and (**b**) S2p core levels. The Mo3d core level result shows three peaks at 232.5, 229.4, and 226.5 eV, which correspond to the $Mo3d_{3/2}$ and $Mo3d_{5/2}$ levels for the Mo3d state and the S2s state, respectively. The S2p core level spectrum exhibits two peaks at 163.3 and 162.2 eV, which are assigned to the doublet, $S2p_{1/2}$ and $S2p_{3/2}$. The calculated stoichiometric ratio is 2 (S/Mo).

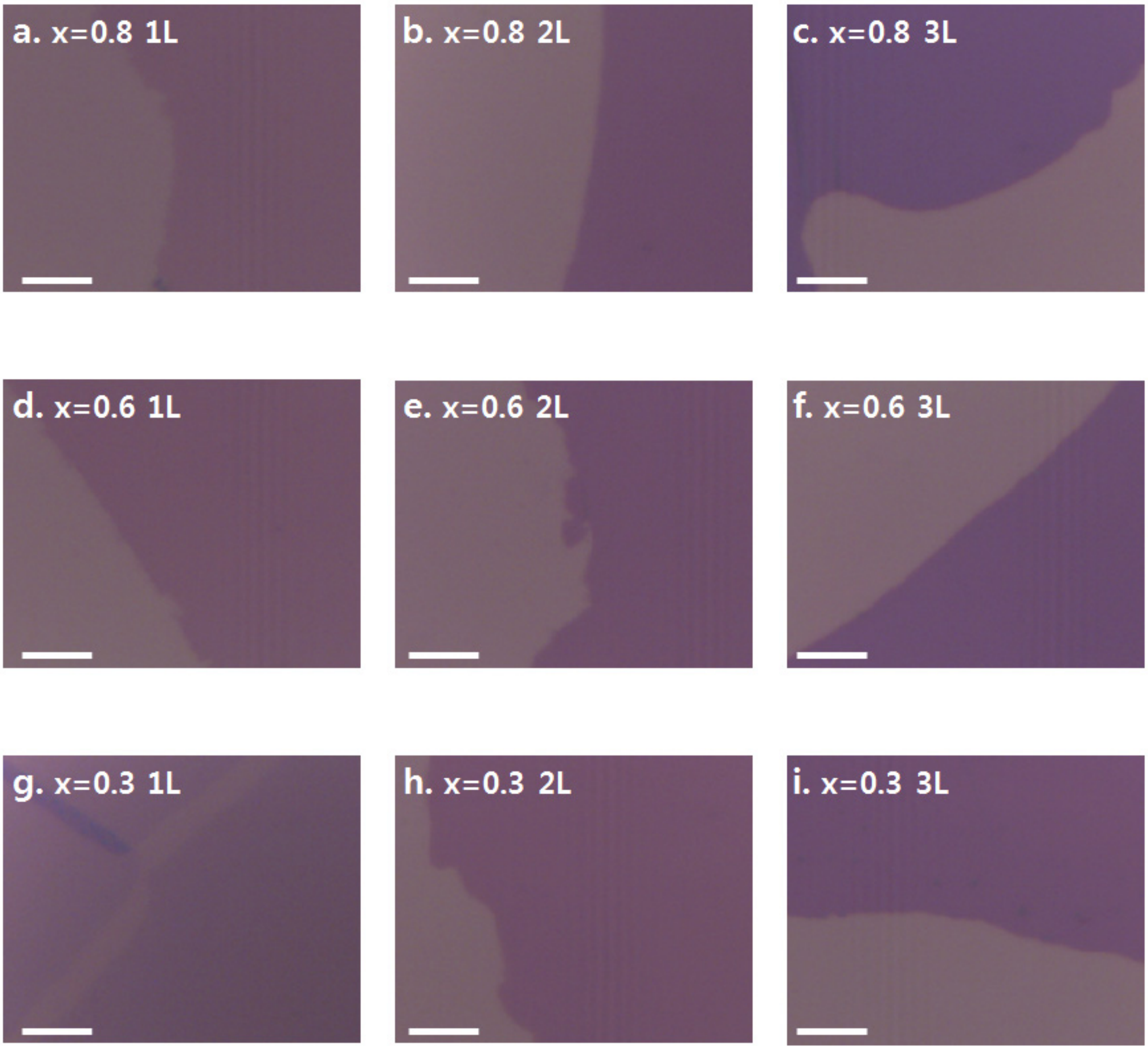

Supplementary Figure 5. OM images of (**a**, **b**, and **c**) 1L, 2L, and 3L $Mo_{0.2}W_{0.8}S_2$, (**d**, **e**, and **f**) 1L, 2L, and 3L $Mo_{0.4}W_{0.6}S_2$, and (**g**, **h**, and **i**) 1L, 2L, and 3L $Mo_{0.7}W_{0.3}S_2$ alloys after transference onto SiO_2 (285 nm) substrate. Scale bars, 5 μm.

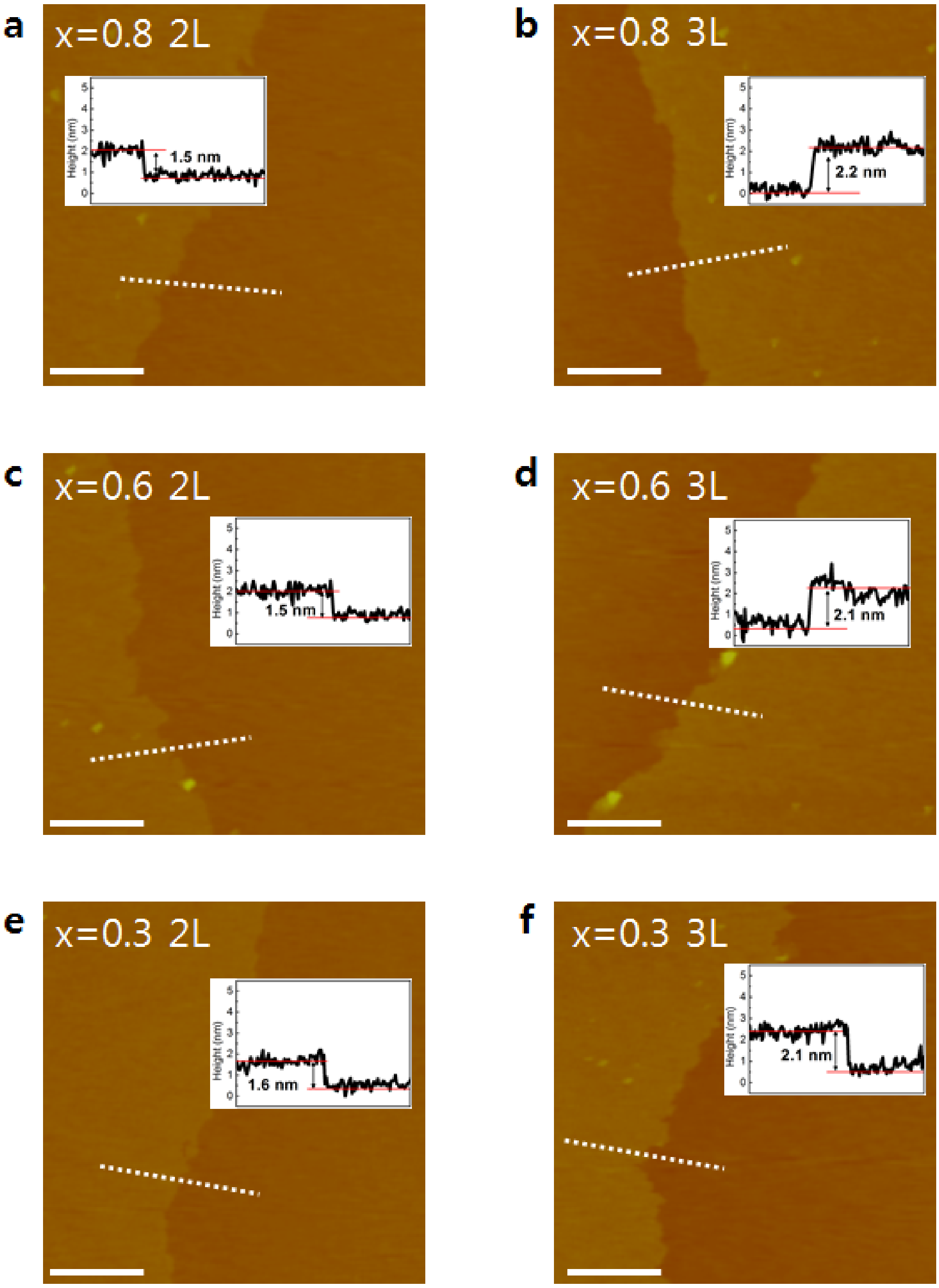

Supplementary Figure 6. AFM images and height profiles for (**a**) 2L and (**b**) 3L $Mo_{0.2}W_{0.8}S_2$, (**c**) 2L and (**d**) 3L $Mo_{0.4}W_{0.6}S_2$, and (**e**) 2L and (**f**) 3L $Mo_{0.7}W_{0.3}S_2$ alloys. Scale bars, 0.5 µm.

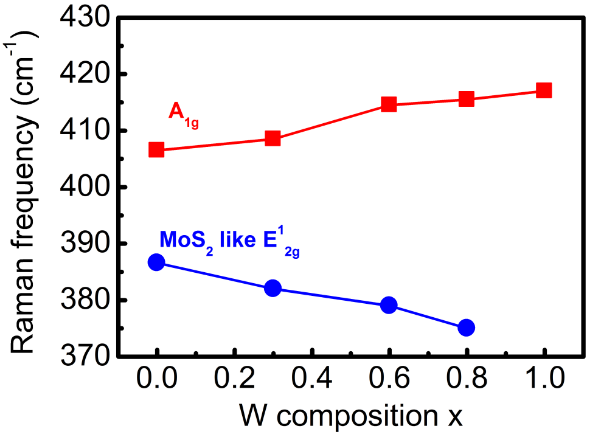

Supplementary Figure 7. Composition-dependent Raman frequencies of A_{1g} and MoS_2-like E^1_{2g} modes of 1L $Mo_{1-x}W_xS_2$ alloys. With an increase in W composition the A_{1g} mode shifts to higher frequency, while the MoS_2-like E^1_{2g} mode downshifts, in agreement with a previous report[2].

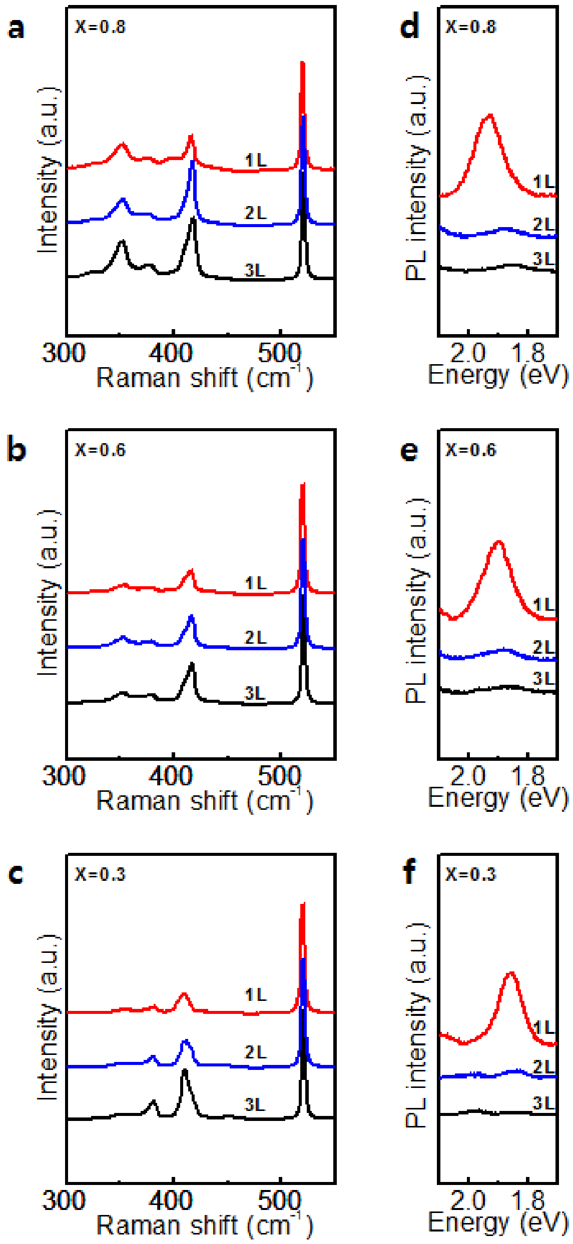

Supplementary Figure 8. (**a**, **b**, and **c**) Raman spectra and (**d**, **e**, and **f**) PL spectra of 1L, 2L, and 3L $Mo_{1-x}W_xS_2$ alloys for x = 0.8, 0.6 and 0.3

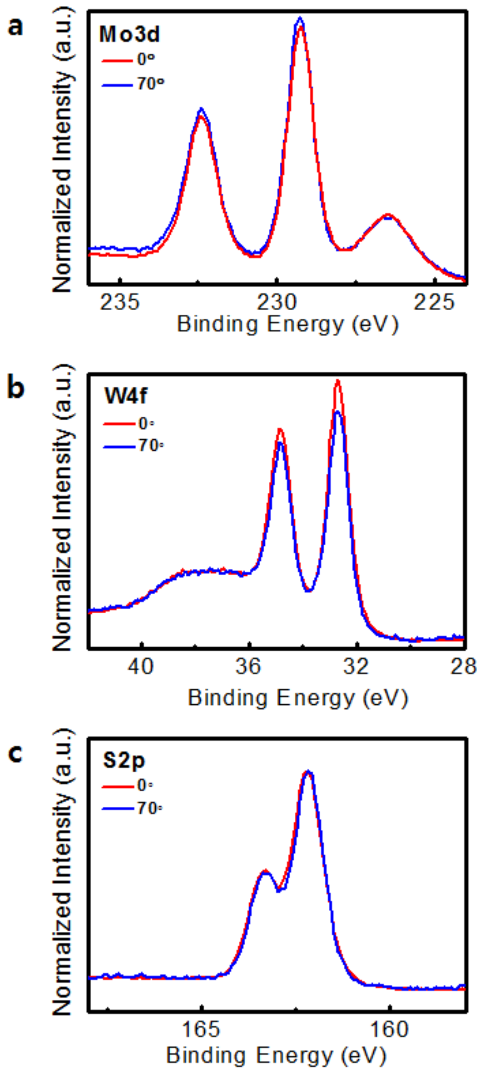

Supplementary Figure 9. ARXPS spectra for (a) Mo3d, (b) W4f, and (c) S2p core levels in a VCC $Mo_{1-x}W_xS_2$ multilayer.

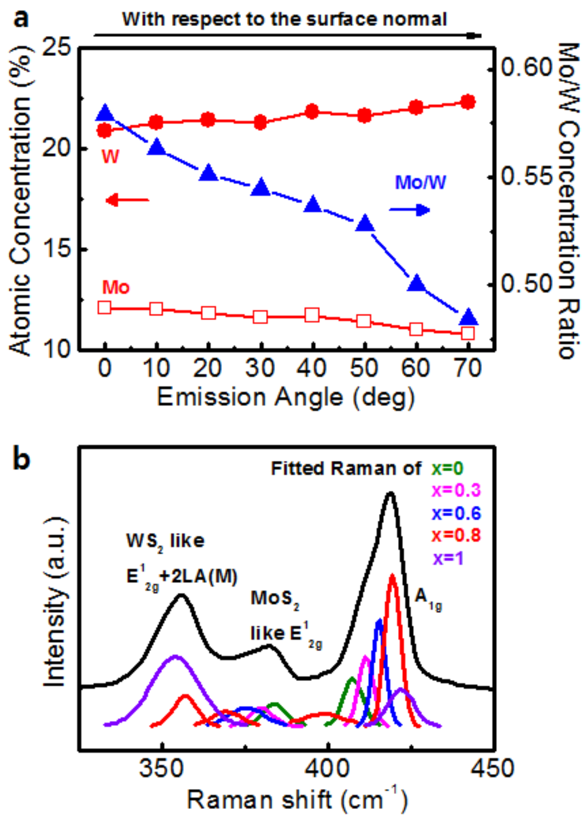

Supplementary Figure 10. (**a**) Calculated the atomic concentration and relative concentration ratio of Mo and W from ARXPS measurement. (**b**) Raman spectra for a VCC $Mo_{1-x}W_xS_2$ multilayer.

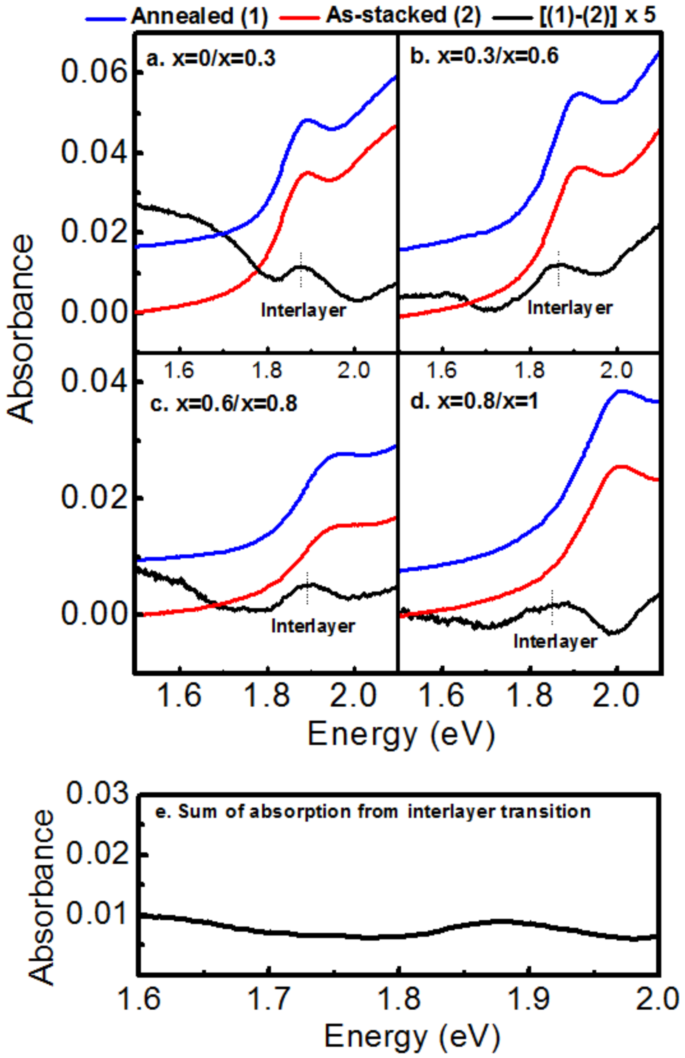

Supplementary Figure 11. Absorption spectra of a stacked VCC $Mo_{1-x}W_xS_2$ multilayer fabricated using the transfer process taken as-transferred (red solid line) and after annealing (blue solid line), and the interlayer transition absorption spectrum, for two different W compositions: (**a**) $x = 0$ and 0.3, (**b**) $x = 0.3$ and 0.6, (**c**) $x = 0.6$ and 0.8, and (**d**) $x = 0.8$ and 1. (**e**) Sum of interlayer transition absorption spectra from Supplementary Figure 11 (a–d). The absorbance peak for the sum of the interlayer transition absorption spectra is at 1.87 eV, which is that same position as that of the stacked VCC $Mo_{1-x}W_xS_2$ multilayer containing 5 different 1L $Mo_{1-x}W_xS_2$ alloys. As a result, we concluded that the absorbance peak at 1.87 eV in Figure 7(c) is attributable to the sum of the absorption peaks from each interlayer transition in the stacked VCC $Mo_{1-x}W_xS_2$ multilayer.

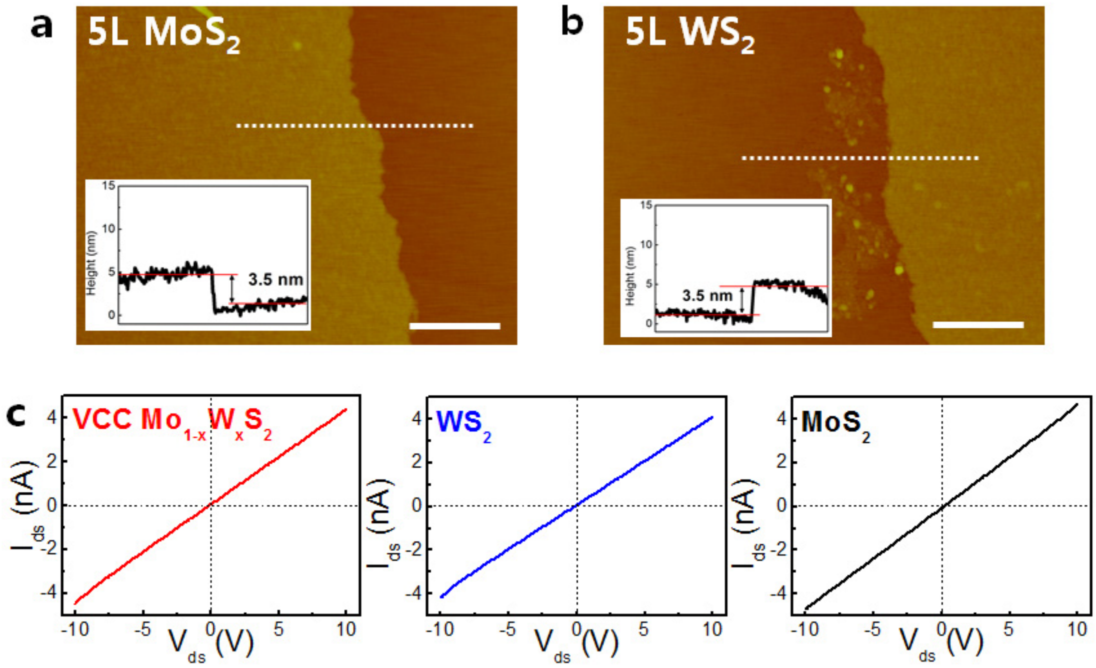

Supplementary Figure 12. AFM images and height profile of transferred 5L (**a**) MoS_2 and (**b**) WS_2. Scale bars, 0.5 μm. (**c**) I-V Characteristics of Photodetectors.

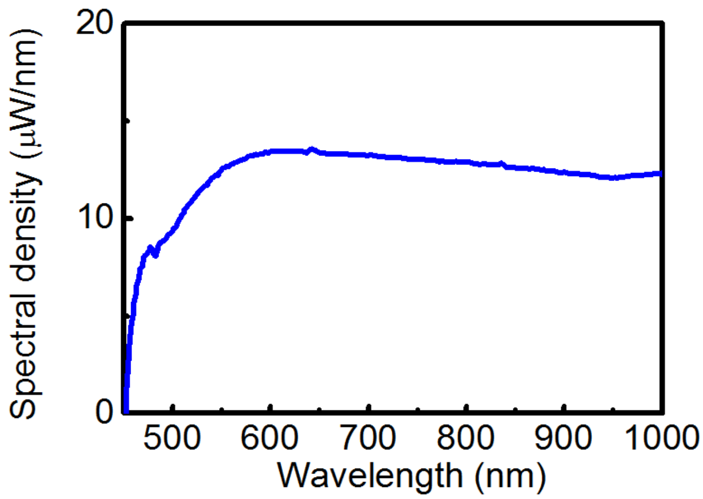

Supplementary Figure 13. Continuum power spectral density depending on energy of incident laser in Figure 7(d)

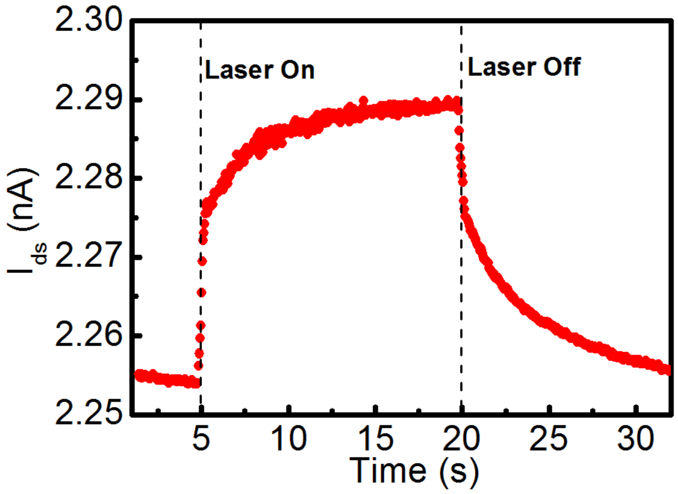

Supplementary Figure 14. Measured time-resolved photocurrent for a VCC $Mo_{1-x}W_xS_2$ multilayer photodetector using specific laser wavelength of 650 nm at V_{ds} = 5 V. The response is characterized by a typical rise time of 4.5 s and a decay time of 7 s, which are corresponding to the single crystal 1L MoS_2 photodetector[3]. This indicates that defect in a VCC $Mo_{1-x}W_xS_2$ multilayer does not have a significant effect on dynamics of photodetector

Supplementary Table 1. Expected Thicknesses of ALD $Mo_{1-x}W_xO_y$ Thin Films

One super-cycle		Expected thickness (nm)		
n	***m***	**MoO_x**	**WO_3**	**$Mo_{1-x}W_xO_y$**
**0**	**10**	**0**	**0.9**	**0.9**
**1**	**6**	**0.27**	**0.54**	**0.81**
**2**	**4**	**0.54**	**0.36**	**0.9**
**3**	**1**	**0.81**	**0.09**	**0.9**
**3**	**0**	**0.81**	**0**	**0.81**

Supplementary Table 2. Scofield Relative Sensitivity Factor for calculation of stoichiometry

**Peak**	**Mo 3d$_{3/2}$**	**Mo 3d$_{5/2}$**	**W 4f$_{5/2}$**	**W 4f$_{7/2}$**	**S 2p$_{1/2}$**	**S 2p$_{3/2}$**
**RSF**	**3.88**	**5.62**	**4.32**	**5.48**	**0.553**	**1.107**

Supplementary References

1. Song, J.-G. et al. Layer-Controlled, Wafer-Scale, and Conformal Synthesis of Tungsten Disulfide Nanosheets Using Atomic Layer Deposition. *ACS Nano*. **7,** 11333-11340 (2013).
2. Chen, Y. et al. Composition-Dependent Raman Modes of $Mo_{1-x}W_xS_2$ Monolayer Alloys. *Nanoscale.* **6,** 2833-2839 (2014).
3. Lopez-Sanchez, O., et al., Ultrasensitive photodetectors based on monolayer MoS_2. *Nature nanotechnology*, 8, 497-501 (2013)
